# The impact of medication reviews by general practitioners on psychotropic drug use and behavioral and psychological symptoms in home-dwelling people with dementia: results from the multicomponent cluster randomized controlled LIVE@Home.Path trial

**DOI:** 10.1186/s12916-022-02382-5

**Published:** 2022-05-26

**Authors:** Marie H. Gedde, Bettina S. Husebo, Janne Mannseth, Mala Naik, Geir Selbaek, Maarja Vislapuu, Line Iden Berge

**Affiliations:** 1 Centre for Elderly and Nursing Home Medicine, Department of Global Public Health and Primary Care, Faculty of Medicine, University of Bergen, Bergen, Norway; 2 Haraldsplass Deaconess Hospital, Bergen, Norway; 3 Municipality of Bergen, Bergen, Norway; 4 Section for Epidemiology and Medical Statistic, Department of Global Public Health and Primary Care, Faculty of Medicine, University of Bergen, Bergen, Norway; 5 Department of Clinical Science, Faculty of Medicine, University of Bergen, Bergen, Norway; 6 Norwegian National Centre for Ageing and Health, Vestfold Hospital Trust, Tønsberg, Norway; 7 Institute of Clinical Medicine, Faculty of Medicine, University of Oslo, Oslo, Norway; 8 Geriatric Department, Oslo University Hospital, Oslo, Norway; 9 NKS Olaviken Gerontopsychiatric Hospital, Askøy, Norway

**Keywords:** Medication review, Deprescribing, Multicomponent intervention, Psychotropic drugs, Behavioral and psychological symptoms of dementia, Neuropsychiatric symptoms, Dementia, Home-dwelling, LIVE@Home.Path

## Abstract

**Background:**

There is limited knowledge regarding the process of deprescribing psychotropic drugs to people with dementia (PwD) conducted by general practitioners (GP). We investigated the impact of a multicomponent intervention, emphasizing medication reviews, on psychotropic drugs and behavioral and psychological symptoms (BPSD) in home-dwelling PwD and quantified change in patient-GP communication evaluated by their informal caregivers.

**Methods:**

LIVE@Home.Path is a stepped-wedge closed-cohort cluster randomized controlled trial for people with mild to moderate dementia aged ≥65 and their informal caregivers (dyads) in Norway. Complementary to health care as usual (control condition), municipal coordinators implemented the multicomponent LIVE intervention: Learning, Innovation, Volunteer support, and Empowerment (including medication review by the PwD’s regular GPs). Block-randomization was used to allocate dyads in three groups receiving the intervention sequentially in periods of 6 months duration. Prepandemic data from the first period is reported, resulting in a 1:2 intervention-to-control ratio. Primary outcome was change in psychotropic drug use. Secondary outcomes were changes in BPSD by Neuropsychiatric Inventory and Cornell Scale of Depression in Dementia and patient-GP communication by an adaption of the Clinical Global Impression of Change.

**Results:**

Four hundred thirty-eight dyads were screened, 280 included, and 237 participated at 6 months (intervention group *n*=67; control condition *n*=170). At baseline, 63% used psychotropic medication regularly: antidementia drugs (47%), antidepressants (13%), hypnotics/sedatives (13%), antipsychotics (5%), and anxiolytics (2%). At 6 months, medication reviews were more frequently conducted in the intervention group compared to control (66% vs 42%, *P*=0.001). We found no differences regarding a change in drug use and BPSD. Patient-GP communication enhanced in the intervention group (mean score 0.95 [standard deviation 1.68] vs 0.41 [1.34], *P*=0.022). In the intervention group, control group, and overall sample, the informal caregivers of those who had their medications reviewed reported improved patient-GP communication compared to those who did not.

**Conclusions:**

Change in psychotropic drug use and BPSD did not differ, even though patient-GP communication improved with medication reviews. Restricted psychotropic drug use among PwD likely reflects more judicious prescribing practices in recent years. Nevertheless, medication reviews could be cultivated to optimize pharmacologic treatment for this complex population.

**Trial registration:**

ClinicalTrials.gov: NCT04043364; registered 15/03/2019.

**Supplementary Information:**

The online version contains supplementary material available at 10.1186/s12916-022-02382-5.

## Background

The number of people with dementia (PwD) is growing dramatically, and the increased disease burden is impacting health care services and societies worldwide [1]. Dementia is a chronic syndrome characterized by progressive cognitive impairments that interfere with daily living, usually accompanied by behavioral and psychological symptoms (BPSD) [1, 2]. BPSD consist of changes in behavior, mood, thoughts, and perception that can be very stressful for the individual and their informal caregivers (family members) [2]. Furthermore, BPSD are associated with poorer cognitive and everyday functioning, which can increase the risk of early transfer from home to permanent nursing home care and reduce life expectancy [3, 4].

Non-pharmacological interventions are recommended as the first-line approach to target BPSD [5, 6]. Although the effects of antipsychotics, anxiolytics, hypnotics and sedatives, antidepressants, and antidementia drugs are modest, these medications may be relegated as a second-line treatment when severe symptoms persist [5, 6]. Moreover, psychotropic drugs may increase the risk of functional decline, strokes, falls, and even early death in this population [7, 8].

The combination and long-term use of these drugs warrant special attention. In a population-based sample from England (*n*=27,090), Richardson et al. (2020) documented that PwD prescribed with Z-hypnotics were more likely to also receive antipsychotics and antidepressants [9]. Similarly, an Italian registry study (*n*=24,735) demonstrated that community-dwelling PwD using antidepressants or antidementia drugs had higher odds of being prescribed antipsychotic medication [10]. Even more, 44% of those receiving antipsychotics were treated longer than was recommended by guidelines [10]. Another registry study from Norway (*n*=22,119) found that indications for use and in-home medication routines for elderly were seldom revised as large-quantum packages of sedatives and hypnotics were frequently issued by general practitioners (GPs) during indirect patient contacts (e.g., office-visit without consultation with the GP or contact by telephone) [11]. However, this study did not specify if the participants were diagnosed with dementia [11]. Data from the REDIC-NH study, collected in Norway between 2012 and 2014, revealed that 68% of PwD (*n*=696) used at least one psychotropic drug at nursing home admission [12]. These consisted of antipsychotics 14%, anxiolytics 17%, hypnotics and sedatives 22%, antidepressants 31%, and antidementia drugs 31% [12]. The frequent use at nursing home admission underlines the need to evaluate the ongoing use of psychotropic drugs in PwD while still residing at home [12]. This is particularly important as approximately 70% of the PwD in Norway are home-dwelling [13].

A recent expert opinion concludes that the next step in the deprescribing field should tailor interventions for home-dwelling PwD while also involving their informal caregivers to identify preferences for medication use and overall health [14]. Such interventions might be considered complex due to the permitted degree of tailoring or inherent properties of the intervention (e.g., multiple and interacting components) [15]. Even though complex interventions are essential for changing clinical practices [15], the best evidence to support deprescribing is for high-risk medications among PwD living in long-term care facilities [14]. For instance, the WHELD trial demonstrated that antipsychotic drug withdrawal was most beneficial for BPSD and mortality for PwD living in nursing homes when social interactions were promoted in parallel [16]. Similarly, physician-led medication reviews embedded in the multicomponent COSMOS trial reduced psychotropic drug use without compromising BPSD, and additionally improved communication between health personnel, nursing home patients, and their relatives [17, 18]. Additionally, communication is an integral part of the work of all Norwegian general practitioners (GPs) in providing continuity in medical care to their enlisted home-dwelling patients. On indication, GPs are obliged to conduct medication reviews among PwD every 6–12 months [6]. Still, we lack knowledge on to which extent they consistently review their medications, as well as the impact of medication reviews on psychotropic drug use. In this substudy, we investigate the impact of a multicomponent intervention emphasizing medication review on changes in psychotropic drug use and BPSD in home-dwelling PwD and their communication with their GPs.

We hypothesize that:

1. The multicomponent intervention emphasizing GP conducted medication reviews will reduce psychotropic drug use.
2. This deprescribing process will not change BPSD but improve patient-GP communication.

## Methods

### Design

**Fig. 1:**
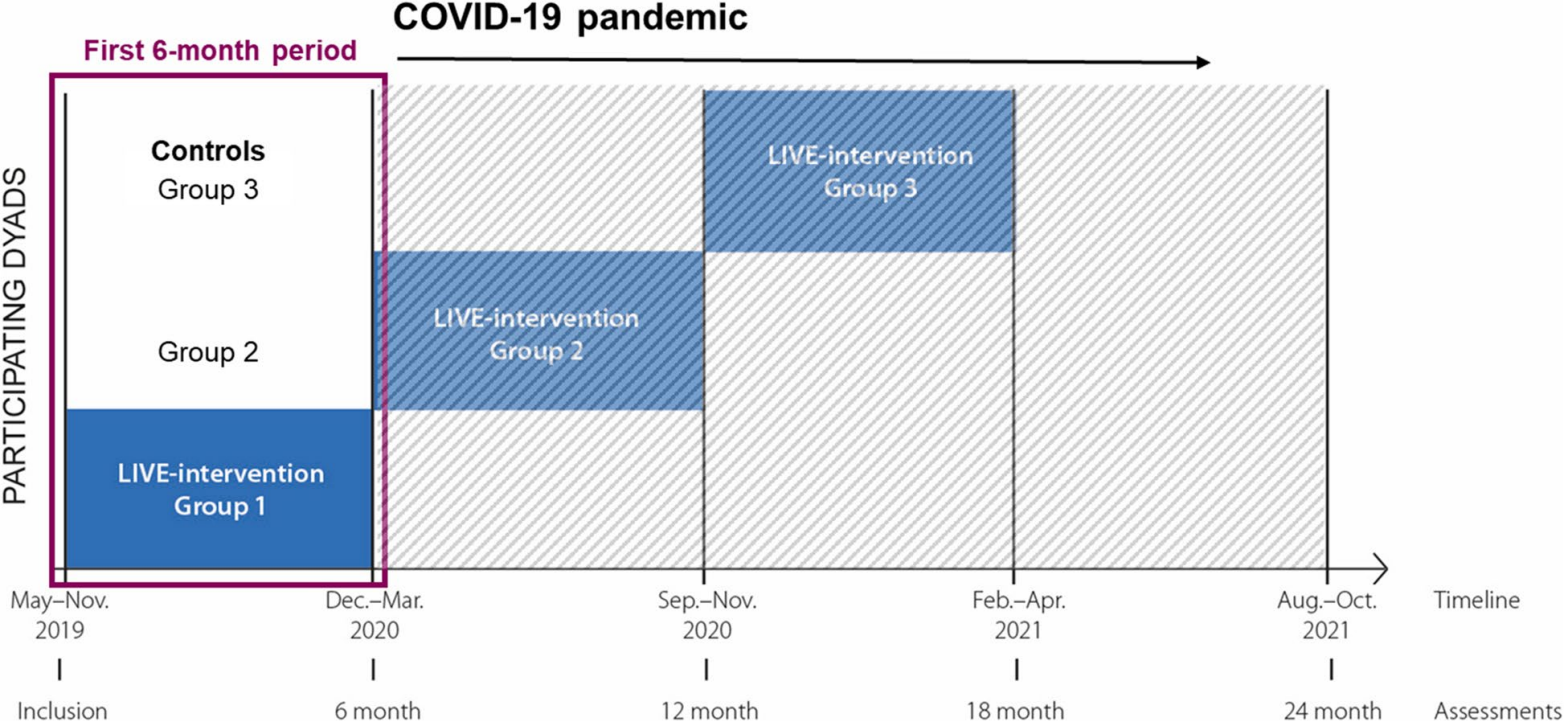
The stepped-wedge closed-cohort randomized controlled LIVE@Home.Path trial during the COVID-19 pandemic. The COVID-19 pandemic temporarily halted the trial protocol at 6 months. This substudy includes all dyads (people with dementia and informal caregivers) who completed the first 6-month period, solely analyzing prepandemic data. In this first 6-month period, dyads randomized to Group 1 received the LIVE (*L*earning, *I*nnovation, *V*olunteer support, and *E*mpowerment) intervention while dyads randomized to Group 2 and Group 3 served as controls receiving health care as usual

**Fig. 2:**
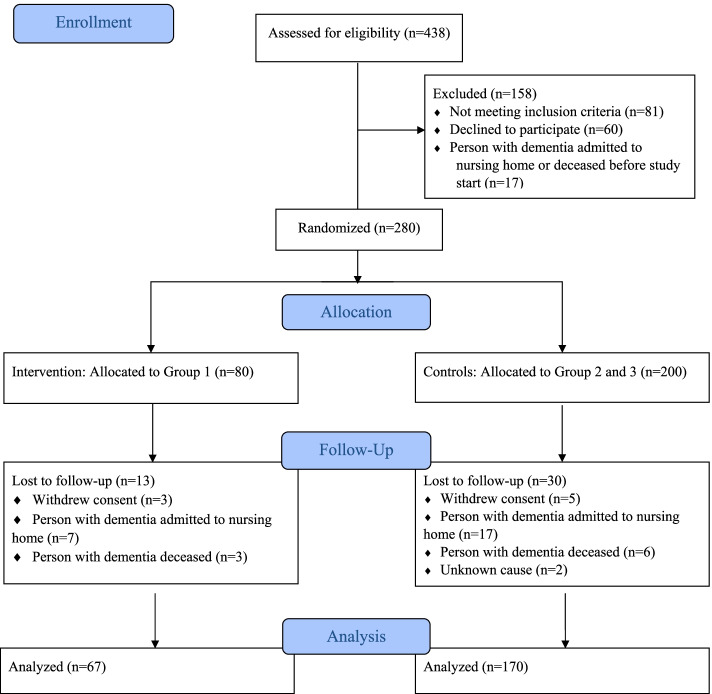
Flow diagram. Dyad (people with dementia and informal caregivers, *n*) flow during the first 6-month period of the LIVE@Home.Path trial

This is a substudy of LIVE@Home.Path: a multicenter, stepped-wedge cluster randomized controlled trial investigating if a multicomponent intervention for dyads of home-dwelling PwD and informal caregivers (family members) improves resource utilization and caregiver burden in dementia care [19]. With 80% power and 5% significance level allowing for 20% loss to follow-up, a sample of 315 dyads was required to detect a difference of 7 care hours per week for the primary outcome care time assessed with Resource Utilization in Dementia [20], based on the assumption that the informal caregivers provided 46 care hours weekly [21]. This stepped-wedge trial used a closed-cohort design, implying that all dyads were recruited before randomization [22]. We used block randomization to allocate dyads in three intervention groups (Group 1, Group 2, Group 3), which were scheduled to receive the multicomponent intervention sequentially in periods of 6 months duration during the 24-month trial (Fig. 1). While the intervention groups were waiting to receive the intervention, they served as controls receiving health care as usual. Dyads were blinded to allocation until their designated coordinator contacted them to receive the intervention, while the nature of the intervention prevented blinding of care providers and dyads. The trial was conducted in Bergen, Bærum, and Kristiansand municipality, Norway, 2019–2021. The first 6-month period was completed in March 2020 as the COVID-19 pandemic temporarily halted the trial protocol (Fig. 1) [23]. Therefore, this substudy includes all dyads completing the first 6-month period, the dyads randomized to Group 1 constitute the intervention group and the dyads randomized to Groups 2 and 3 constitute the control group (Fig. 2).

### Intervention

LIVE is an acronym for the multicomponent intervention in which a coordinator facilitated Learning, Innovation, Volunteer support, and Empowerment emphasizing medication reviews. Table 1 outlines the intervention components, while we refer to the trial protocol for a full description that also covers the implementation process in detail [19]. The multicomponent intervention was developed using the theoretical framework by the UK Medical Research Council on complex interventions [15]. The intervention was designed to meet the requirements of the Dementia Plan 2020 by the Royal Norwegian Ministry of Health and Care Services, combining and adapting already existing evidence on how to support PwD [24, 25].

**Table 1:** The multicomponent LIVE intervention implemented during the 6-month intervention period of the LIVE@Home.Path trial

	Learning	Innovation	Volunteer support	Empowerment
Content	Learning programs on dementia -Etiology, symptoms and disease course -Legal rights -Safety -Economy -Coping	Assess the need for, evaluate the usefulness of, and inform about relevant assistive technology and telecare -Passive sensors -Active sensors and tracking devices -Everyday technology -Video communication	Explore attitudes towards volunteer services and initiate contact with non-profit organizations -The Red Cross -Norwegian Association of Public Health	Establish contact with the regular general practitioner to initiate: -Advanced Care Planning -Medication review
Participants	-PwD -Informal caregiver -Coordinator	-PwD -Informal caregiver -Coordinator	-PwD -Informal caregiver -Coordinator -Volunteers from nonprofit organizations matched by volunteer managers	-PwD -Informal caregiver -Coordinator -PwD’s regular general practitioner

Each component of the intervention was implemented by a municipal coordinator

*PwD* people with dementia

The coordinators were nurses, learning disability nurses, and occupational therapists experienced in dementia care already working in the home-based services of the designated municipalities. In the intervention period, each coordinator served approximately 5–7 dyads in addition to other municipal tasks not affiliated with the trial. The research group held two-day implementation seminars at the start of the intervention period to qualify the coordinators to adapt the intervention to the dyad’s needs through lectures, role-plays, and discussions. Pocket manuals describing core features of the intervention guided coordinators in addressing all the intervention components. The coordinators used checklists to document to which extent they had introduced the dyads to the intervention components. To further standardize and secure implementation, we arranged one-day midway seminars halfway through the 6-month intervention period allowing for discussion of obstacles and pitfalls, and telephone follow-up for the coordinators every 14 days*.*

The coordinators paid the dyads at least two home visits and made monthly telephone calls during the 6-month intervention period. They provided the dyads with verbal and written information on the intervention components in the context of their municipality (Table 1) and established contact with the PwD’s regular GP to inform on participation. If welcomed by the dyads, the coordinators requested a medication review directly from the PwD’s regular GP using the electronic medical record and provided a report on BPSD, cognition, blood pressure, pulse, body mass index, pain, and caregiver burden (Relative Stress Scale) prior to the in-person consultation [19, 26]. The GPs evaluated the indication for medication reviews based on the report, medical history, and relevant laboratory tests. The informal caregivers and coordinators were encouraged to partake in the medication review in addition to the PwD to acquire a better understanding of the current symptoms and complaints, and to empower the PwD in discussing the use of medications and any wishes for treatment. The GPs were responsible for and made all final decisions regarding the PwD’s medical treatment. Additional file 1 outlines the role of health care professionals involved in the conduction of LIVE@Home.Path trial.

### Participants

We applied convenience sampling to recruit dyads from geriatric and gerontopsychiatric out-patient clinics, municipal memory teams, and general media with no financial incentives. Dyads were eligible if the PwD was ≥65 years, home-dwelling, and in face-to-face contact with the informal caregiver at least 1 h a week. Dementia, as diagnosed by the health care services, qualified individuals for participation regardless of etiology as long as their Mini-Mental Status Examination (MMSE) score was 15–26 or Functional Assessment Staging (FAST) score was 3–7 [19, 23, 27, 28]. A dyad was lost at follow-up if consents were withdrawn or if the PwD was permanently admitted to a nursing home or deceased.

### Assessments and outcomes

The data collectors at municipal sites (nurses, learning disability nurses, occupational therapists) completed a one-day training program arranged by the research team to safeguard blinded and standardized data collection. Instructions were given both verbally and in writing. The researchers were available for answering any questions regarding the assessments and provided technical support, as well as assistance, during data collection. Data were immediately transferred to a secure server using tablets.

#### Primary outcomes

Changes in the numbers of prescribed psychotropic drugs, both in total and regular use, were calculated from baseline to month 6. The dyads reported all the prescription and over-the-counter medicines and supplements the PwD was currently using. The information was confirmed from prescriptions, drug packages, multi-dose drug dispensing, and/or medical records. All substances listed in the Anatomical Therapeutic Chemical Index (ATC) were classified as drugs [29]. The identity of the drugs was split, with those drugs set in a schedule regarded as “regular” and all others “on-demand.” Psychotropic drugs were categorized according to ATC in antipsychotic (N05A), anxiolytic (N05B), hypnotic and sedative (N05C), antidepressant (N06A), and antidementia drugs (N06D).

#### Secondary outcomes

The Neuropsychiatric Inventory (NPI-12) was used to evaluate delusions, hallucinations, agitation, depression, anxiety, euphoria, apathy, disinhibitions, irritability, aberrant motor behavior, sleep disturbances, and appetite changes over the four preceding weeks at baseline and 6 months [30]. Multiplying frequency (1–4) and severity (1–3) generated a score for each of the twelve domains, with domain scores ≥4 indicating symptoms of clinical relevance [2]. Domain scores were added to yield the NPI-12 total score (0–144). According to a previous principal component analysis, subsyndrome scores for psychosis comprised delusions and hallucinations (0–24), hyperactive behavior included agitation, euphoria, irritation, disinhibition, and aberrant motor behavior (0–60), while depression, apathy, sleep disturbances, and appetite changes constituted the mood subsyndrome (0–48) [31].

The Cornell Scale for Depression in Dementia (CSDD) assessed the depressive symptoms of the past week at baseline and 6 months [32]. The nineteen items were rated “absent” (0), “mild or intermittent” (1) or “severe” (2), or “not possible to evaluate” (missing); these were then added to generate the CSDD total score (0–38). The CSDD total score ≥8 indicated depressive symptoms of clinical relevance [33]. NPI-12 and CSDD were selected due to robust psychometric properties [30, 32–34].

The Clinical Global Impression of Change (CGIC) [35] was adapted to measure meaningful change in communication with the PwD’s regular GP as perceived by the informal caregivers. At six months, change compared to baseline was rated on a 11-point scale from − 5 “Very much worse” via 0 “No change” to 5 “Very much improved.” A similar formulation has been applied in nursing homes [36].

#### Characteristics

At baseline, dementia etiology was classified following the International Classification of Diseases (ICD-10) [37], while MMSE (range: 0–30, a lower score indicates greater cognitive impairment) and FAST (range: 1–7, a higher score indicates lesser functioning) covered dementia severity [27, 28]. Dependency of daily living was assessed by Physical Self-Maintenance Scale (PSMS, range: 6–30) and Instrumental Activities of Daily Living Scale (IADL, range: 8–31), in which higher scores indicate higher dependency [38, 39]. Medical comorbidity was evaluated by the one-item General Medical Health Rating Scale (GMHR) as poor, fair, good, or excellent health [40]. Data on kinship, age, gender, and residency within the dyads were also registered. At 6 months, the dyads reported whether the GP had reviewed the PwD’s medications in the preceding 6 months.

### Statistical methods

The unequal variances *t*-test was used to compare the intervention to the control group by changes in 1.) psychotropic drug use and BPSD between time points and 2.) patient-GP communication. Pearson’s chi-squared test was used to evaluate to what extent medication reviews were conducted (reach) as well as determine the attrition rates between groups. Subgroup analyses comparing 1.) characteristics across the intervention and control group, 2.) those who had their medications reviewed to those who did not within (a) the intervention and (b) control groups, and 3.) completers and non-completers were made at baseline using Pearson’s chi-square test for categorical data, the unequal variances t-test for normally distributed data, and the Wilcoxon-Mann-Whitney test for non-normal data. Characteristics are presented by number (*n*) and percent; mean and standard deviation (SD); and median and interquartile range (IQR), respectively. Two-tailed *P* values <0.05 were regarded as significant. NPI-12 and CSDD total scores were generated without substitution when >80% of the instruments were answered by the informal caregivers. Otherwise, they were regarded as missing. For all data, missing ranged from 0 to 6% (CSDD total score at baseline). We performed all analyses with Stata/IC, release 17 (StataCorp LP, College Station, TX).

## Results

Of the 438 dyads screened for participation in LIVE@Home.Path, 280 dyads were included from May to November 2019 (Fig. 2). Table 2 presents baseline characteristics for the 237 dyads still in study at 6 months, 67 of which received the intervention. Alzheimer’s disease was the dementia etiology most frequently specified (*n*=86, 36%). Antidementia drugs were the most frequently used psychotropic drug class, being regularly prescribed to 112 (47%) PwD. Psychotropic drugs, apart from antidementia drugs, were regularly prescribed to 69 (29%) PwD, and 12 (5%) used two or more. The median NPI-12 total score was 12 (IQR 4 to 24), and 159 PwD (67%) displayed one domain or more of clinical relevance. Mood was the NPI-12 subsyndrome with the highest median score, namely 4.5 (IQR 0 to 11). The median CSDD total score was 5 (IQR 1 to 9*)*, and 73 (31%) of the overall sample suffered from depressive symptoms of clinical relevance.

**Table 2:** Baseline characteristics for people with dementia and informal caregivers in the LIVE@Home.Path trial

	Overall sample (*n*=237)		Intervention group (Group 1) (*n*=67)		Controls (Group 2 and 3) (*n*=170)		*P* value*
	*n* (%)	Mean (SD)/median [IQR]	*n* (%)	Mean (SD)/median [IQR]	*n* (%)	Mean (SD)/median [IQR]	
**Person with dementia**							
Age		82 (7)		83 (7)		81 (7)	0.013*
Gender, female	149 (63)		46 (69)		103 (61)		0.268
Residency							0.657
Living alone	102 (43)		32 (48)		70 (41)		
Co-residing with the reporting caregiver	111 (47)		29 (43)		82 (48)		
Co-residing with someone else than the reporting caregiver	20 (8)		5 (7)		15 (9)		
Dementia etiology							0.207
Alzheimer’s disease	86 (36)		22 (33)		64 (38)		
Vascular dementia	7 (3)		0 (0)		7 (4)		
Dementia in other diseases classified elsewhere	11 (5)		2 (3)		9 (5)		
Unspecified dementia	131 (55)		42 (63)		89 (52)		
MMSE		21 [18, 23]		21 [19, 24]		21 [17, 23]	0.295
FAST		4 [4, 4]		4 [4, 4.5]		4 [4, 4]	0.064
GMHR							0.026*
Poor health	5 (2)		0 (0)		5 (3)		
Fair health	74 (31)		30 (45)		44 (26)		
Good health	110 (48)		26 (39)		84 (49)		
Excellent health	40 (17)		9 (13)		31 (18)		
PSMS		10 [8, 12]		10 [8, 13]		10 [8, 11]	0.146
IADL		20 [15, 25]		20 [15, 25]		20 [15, 24]	0.566
Drugs in general							
Total number	221 (93)	5 [3, 7]	63 (94)	5 [4, 7]	158 (93)	5 [3, 7]	0.633^#^
Regularly	219 (92)	5 [3, 7]	62 (93)	5 [3, 7]	157 (92)	5 [3, 7]	0.810^#^
Psychotropic drugs							
Total number	159 (67)	1 [0, 1]	49 (73)	1 [1, 1]	110 (65)	1 [0, 1]	0.379^#^
Regularly	150 (63)	1 [0, 1]	44 (66)	1 [0, 1]	106 (62)	1 [0, 1]	0.870^#^
Antipsychotic drugs	11 (5)		4 (6)		7 (4)		
Anxiolytic drugs	5 (2)		2 (3)		3 (2)		
Hypnotic/sedative drugs	31 (13)		8 (12)		23 (14)		
Antidepressant drugs	31 (13)		8 (12)		23 (14)		
Antidementia drugs	112 (47)		32 (48)		80 (47)		
Regularly psychotropic drugs except for antidementia drugs	69 (29)	0 [0, 1]	20 (30)	0 [0, 1]	49 (29)	0 [0, 1]	0.970^#^
Concomitant use of psychotropic drugs except for antidementia drugs	12 (5)		2 (3)		10 (6)		
On-demand	17 (7)	0 [0, 1]	7 (10)	0 [0, 1]	10 (6)	0 [0, 1]	0.221^#^
Antipsychotic drugs	1 (0)		0 (0)		1 (1)		
Anxiolytic drugs	8 (3)		2 (3)		6 (4)		
Hypnotic/sedative drugs	9 (4)		6 (9)		3 (2)		
Antidepressant drugs	0 (0)		0 (0)		0 (0)		
Antidementia drugs	0 (0)		0 (0)		0 (0)		
NPI-12 total score		12 [4, 24]		15 [5, 26]		12 [3.5, 20]	0.166
NPI-12 subsyndromes							
Psychosis		0 [0, 2]		0 [0, 2]	0 [0, 2]		0.745
Hyperactive behavior		2 [0, 5]		2 [0, 8]	2 [0, 5]		0.579
Mood		6 [1, 12]		7 [1, 14]	4.5 [0, 11]		0.134
NPI-12 domain scores							
Delusions	37 (16)	0 [0, 2]	8 (12)	0 [0, 1]	29 (17)	0 [0, 2]	0.631^#^
Hallucinations	16 (7)	0 [0, 0]	4 (6)	0 [0, 0]	12 (7)	0 [0, 0]	0.346^#^
Agitation	18 (8)	0 [0, 1]	4 (6)	0 [0, 1]	14 (8)	0 [0, 1]	0.530^#^
Depression	58 (24)	0 [0, 2]	20 (30)	1 [0, 6]	38 (22)	0 [0, 2]	0.169^#^
Anxiety	42 (18)	0 [0, 2]	16 (24)	0 [0, 2]	26 (15)	0 [0, 1]	0.451^#^
Euphoria	4 (2)	0 [0, 0]	0 (0)	0 [0, 0]	4 (2)	0 [0, 0]	0.718^#^
Apathy	65 (27)	0 [0, 4]	23 (34)	1 [0, 6]	42 (25)	0 [0, 4]	0.133^#^
Disinhibitions	19 (8)	0 [0, 1]	5 (7)	0 [0, 1]	14 (8)	0 [0, 1]	0.991^#^
Irritability	47 (20)	0 [0, 2]	16 (24)	0 [0, 3]	31 (18)	0 [0, 2]	0.574^#^
Aberrant motor behavior	28 (12)	0 [0, 0]	9 (13)	0 [0, 0]	19 (11)	0 [0, 0]	0.542^#^
Sleep disturbances	48 (20)	0 [0, 2]	12 (18)	0 [0, 1]	36 (21)	0 [0, 2]	0.745^#^
Appetite changes	65 (24)	0 [0, 3]	21 (31)	0 [0, 5]	44 (26)	0 [0, 3]	0.989^#^
≥ 1 NPI-12 domain of clinical relevance	159 (67)		49 (67)		110 (65)		0.252
CSDD total score	73 (31)	5 [1, 9]	22 (35)	6 [2, 9]	51 (30)	4.5 [1, 9]	0.573^#^
**Informal caregiver**							
Age		66 (12)		67 (13)		66 (12)	0.749
Gender, Female	152 (64)		44 (66)		108 (64)		0.816
Kinship to the person with dementia							0.765
Spouse	103 (43)		27 (40)		76 (45)		
Child	116 (49)		36 (54)		80 (47)		
Other	13 (5)		3 (4)		10 (6)		

*n* number of participants completing the first 6-month period, *SD* standard deviation, *IQR* interquartile range, *P* two-tailed *P* value, generated by Pearson’s chi-square, unequal variances *t*-test, or Wilcoxon-Mann-Whitney test, regarded significant if <0.05 and marked *, ^#^*P* value of comparison of non-normal or normal data when categorical data also is reported. *MMSE* Mini-Mental Status Examination, range 0–30, a lower score indicates greater impairment; *FAST* Functional Assessment Staging, range 1–7, a higher score indicates lesser functioning; *GMHR* General Medical Health Rating Scale; *PSMS* Physical Self-Maintenance Scale, range 6–30, a higher score indicates higher dependency; *IADL* Instrumental Activities of Daily Living Scale, range 8–31, higher score indicates higher dependency. Drugs were classified by the Anatomical Therapeutic Chemical Index; psychotropic drugs included antipsychotics, anxiolytics, hypnotics/sedatives, antidepressants, and antidementia drugs. *NPI-12* Neuropsychiatric Inventory, total score ranges 0–144, psychosis subsyndrome (delusions and hallucinations) ranges 0–24, hyperactive behavior (agitation, euphoria, irritation, disinhibition, aberrant motor behavior) ranges 0–60, mood (depression, apathy, sleep disturbances, and appetite changes) ranges 0–48, each domain ranges 0–12 with domain scores ≥4 indicating symptoms of clinical relevance; *CSDD* Cornell Scale for Depression in Dementia, total score ranges 0–38 and ≥8 indicate depressive symptoms of clinical relevance

**Fig. 3:**
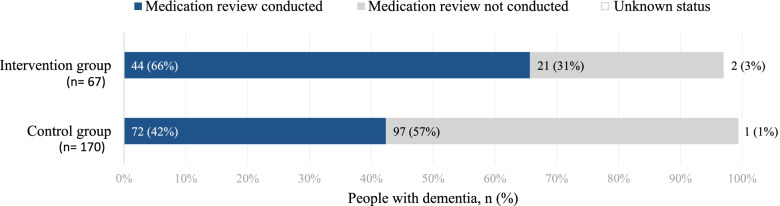
Reach of medication reviews. Conduction of medication reviews for people with dementia (*n* (%)) during the first 6-month period of the LIVE@Home.Path trial

During the 6-month intervention period, GPs reviewed the medications of 44 (66%) PwD in the intervention group and 72 (42%) of the controls (*P*=0.001) (Fig. 3). Within the intervention group, PwD who had their medications reviewed used psychotropic drugs more widely had higher levels of hallucinations and agitation and a lower level of functioning at baseline than their counterparts not receiving medication reviews (Additional file 2). In the control group, the GPs conducted medication reviews more often for women, those with greater cognitive impairments, and those using hypnotics/sedatives (data not shown).

From baseline to six months, changes in the use of psychotropic drugs and individual drug classes did not differ between the intervention and control groups using the unequal variances *t*-test (Table 3). Similarly, Table 3 shows that no differences in NPI-12 or CSDD were detected.

**Table 3:** Changes from baseline to 6 months for people with dementia in the LIVE@Home.Path trial

	Number of observations (overall sample)	Intervention group (Group 1) (*n*=67)		Controls (Group 2 and 3) (*n*=170)		*P* value*
	*n*	Mean	SD	Mean	SD	
**Drugs in general**						
Total number	213	0.32	2.17	0.29	1.94	0.944
Regularly	213	0.02	1.80	− 0.06	1.63	0.778
**Psychotropic drugs**						
Total number	213	0.02	0.81	0.06	0.62	0.718
Regularly	213	0.00	0.64	− 0.01	0.61	0.946
≥ 1 regularly	138	− 0.18	0.60	− 0.12	0.66	0.620
Classes regularly prescribed						
Antipsychotic drugs	213	− 0.02	0.13	0.00	0.00	0.321
Anxiolytic drugs	213	0.00	0.00	0.01	0.18	0.656
Hypnotic/sedative drugs	213	0.02	0.34	− 0.03	0.31	0.337
Antidepressant drugs	213	0.03	0.26	0.02	0.29	0.737
Antidementia drugs	213	− 0.03	0.45	0.00	0.43	0.623
**Behavioral and psychological symptoms of dementia**						
NPI-12 total score	220	2.57	18.60	2.64	16.60	0.982
NPI-12 subsyndromes						
Psychosis	237	0.54	3.73	0.79	4.23	0.647
Hyperactive behavior	237	2.66	7.96	1.34	7.98	0.252
Mood	237	− 0.46	11.15	0.51	9.23	0.527
NPI-12 domain scores						
Delusions	219	0.67	2.47	0.43	2.99	0.599
Hallucinations	219	0.03	2.27	0.35	2.29	0.353
Agitation	218	0.73	2.94	0.45	2.35	0.509
Depression	220	− 0.07	3.76	0.31	2.95	0.479
Anxiety	218	− 0.08	3.02	0.06	3.47	0.761
Euphoria	216	0.52	1.81	0.19	1.74	0.227
Apathy	218	0.03	4.47	0.30	4.01	0.685
Disinhibitions	216	0.32	2.68	− 0.17	2.28	0.219
Irritability	220	0.08	3.72	0.50	3.02	0.431
Aberrant motor behavior	218	1.13	3.57	0.14	3.08	0.059
Sleep disturbances	217	0.42	4.22	0.40	4.23	0.981
Appetite changes	219	− 1.23	4.62	0.35	3.59	0.183
CSDD total score	218	2.12	5.09	0.90	7.69	0.178
**Patient-general practitioner communication by CGIC**	230	0.95	1.68	0.41	1.34	0.022*

*n* number of participants completing the first 6-month period; *SD* standard deviation, *P* two-tailed *P* value, generated by unequal variance *t*-test, regarded significant if <0.05 and marked *. Drugs were classified by the Anatomical Therapeutic Chemical Index; psychotropic drugs included antipsychotics, anxiolytics, hypnotics/sedatives, antidepressants, and antidementia drugs. *NPI-12* Neuropsychiatric Inventory, total score ranges 0–144, psychosis subsyndrome (delusions and hallucinations) ranges 0–24, hyperactive behavior (agitation, euphoria, irritation, disinhibition, aberrant motor behavior) ranges 0–60, mood (depression, apathy, sleep disturbances, and appetite changes) ranges 0–48, each domain ranges 0–12. *CSDD* Cornell Scale for Depression in Dementia, total score ranges 0–38. Negative values indicate reductions in drugs and improvement on NPI and CSDD, while positive scores indicate drug increase and symptom deterioration. *CGIC* Clinical Global Impression of Change, range −5–5, negative scores indicate worsening, positive scores indicate improvement

**Fig. 4:**
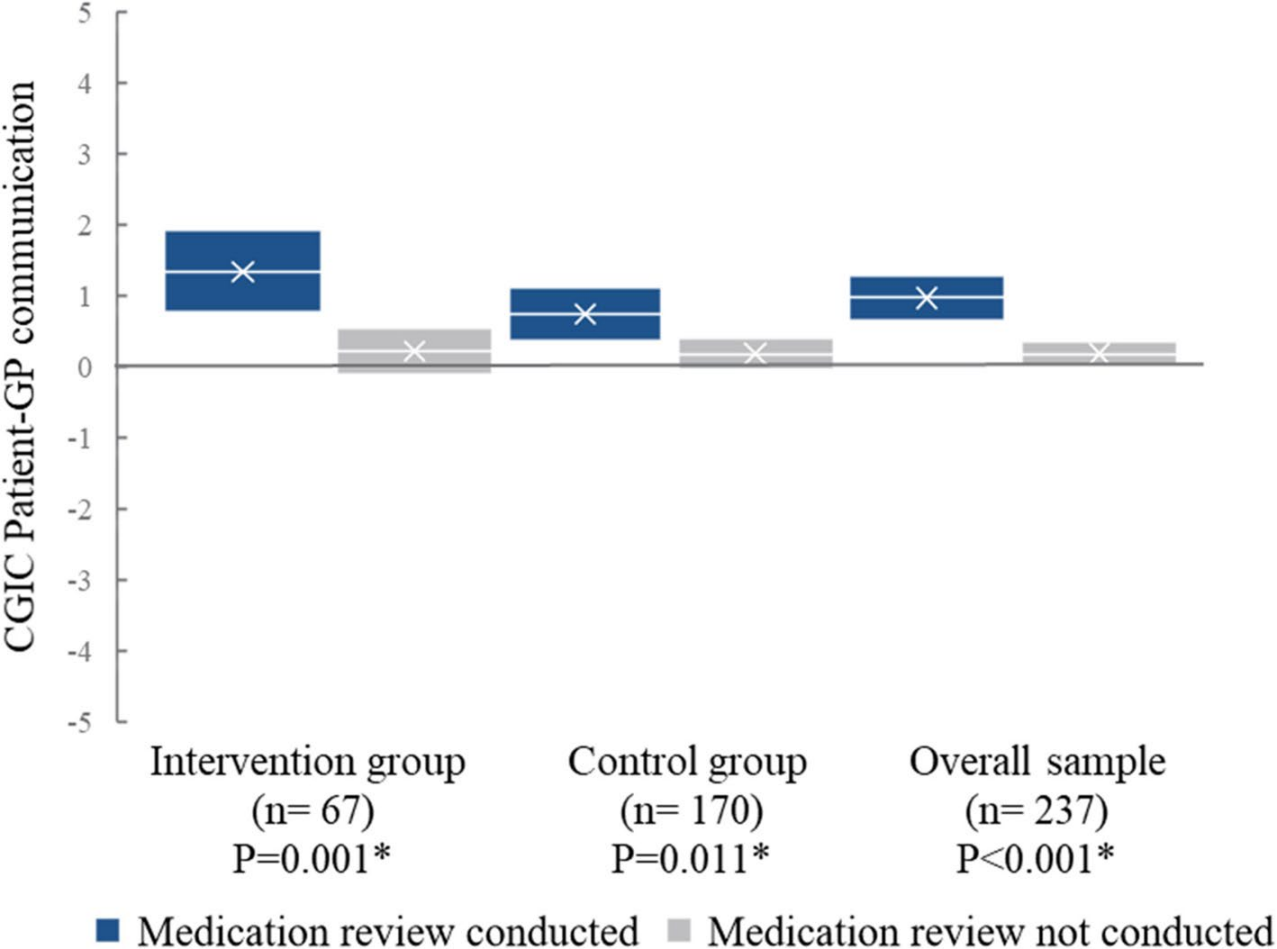
Change in patient-general practitioner (GP) communication by medication reviews. Patient-GP communication as perceived by the informal caregivers stratified on whether medication reviews were conducted for people with dementia (*n*) during the first 6-month period of the LIVE@Home.Path trial. CGIC: Clinical Global Impression of Change, range −5–5, negative value: worsening, positive value: improvement. *P* values for difference in mean, marked * if <0.05, and 95% confidential interval by the unequal variances *t*-test

We found significant intervention effects regarding patient-GP communication (Table 3). The informal caregivers of PwD who had their medications reviewed reported improved patient-GP communication compared to those who did not have a medication review conducted. This was true for the intervention group (1.33 vs. 0.20, *P*=0.001) and control group (0.73 vs. 0.17, *P*=0.011), as well as the overall sample (0.96 vs. 0.17, *P*<0.001) (Fig. 4).

The attrition rates from baseline to 6 months were similar in both groups: 16% in the intervention group and 15% in the control group (*P*=0.793). In most cases, dyads were lost at follow-up because the PwD was permanently admitted to a nursing home or deceased (Fig. 2). The non-completers (*n*=43) were older, had a lower level of functioning by FAST, a higher dependency in daily living activities by PSMS and IADL, and used antidementia drugs less often than the completers (*n*=237) (Additional file 3). We found the same differences when comparing completers (*n*=237) to people lost at follow-up due to permanent nursing home care (*n*=24), the exceptions being higher NPI-12 total score (17.5, IQR 8 to 28.5, vs 12, IQR 4 to 24, *P*=0.036) and the number of NPI-12 domains of clinical relevance (2, IQR 1 to 3.5, vs 1, IQR 0 to 3, *P*=0.027).

## Discussion

The multicomponent intervention of LIVE@Home.Path successfully increased the reach of medication reviews conducted by GPs, yet the process led to no change in psychotropic drug use or BPSD for home-dwelling PwD. Nevertheless, their informal caregivers perceived an improvement in communication with the GP. We argue that our control group serves as an example of an existing practice among Norwegian GPs for optimizing pharmacological BPSD management through medication reviews. Moreover, these established procedures can be even more cultivated, because our study shows that when GPs are encouraged, they increase the reach of revisions for home-dwelling PwD, leading to better communication.

This is the first study to evaluate the impact of GP conducted medication reviews on psychotropic drugs in home-dwelling PwD. Contrary to our primary hypothesis, we detected no impact on prescribing practices, although it was demonstrated that medication reviews reduce the number of psychotropic drugs prescribed in nursing homes [18, 41]. The pre-revision levels of psychotropic drugs used both regularly and on-demand were lower in our study than in the nursing home setting, which might make further reductions uncalled for. This is also illustrated by the German Delphi-MV trial enrolling persons living at home with mild cognitive impairment and dementia (*n*=407), in which interdisciplinary case conferences failed to reduce the number of potentially inappropriate drugs (24%) yet increased the use of antidementia drugs [42]. In a Finnish population-based sample of older adults (*n*=700), in which close to 40% used antipsychotics, benzodiazepines, and antidepressants, geriatricians outside the health care system were not able to reduce psychotropic drug use by structural medication assessments [43]. This reflects that deprescribing is challenging even for highly specialized physicians in populations with prevalent use. Nevertheless, the authors emphasized the potential of medication reviews in preventing psychotropic polypharmacy, above all in continuous patient-physician relationships allowing for careful considerations also before initiating new drugs [43]. In Norway, the cluster randomized controlled COOP trial confirmed this view, concluding that even though regular GPs were less experienced than geriatricians in performing structured evaluations of complex pharmacotherapy, they contributed to collaborative medication reviews with valuable input as they knew their patients well [44]. A recent retrospective cohort study with a 1-year follow-up on 9324 patients with dementia in England concluded that higher continuity of GP care was associated with safer prescribing and lower rates of major adverse events [45]. Another retrospective study including 2250 new residents with dementia found that psychotropic drugs were dispensed at higher rates for those who changed GP when entering Australian residential care compared to those who continued seeing their regular GP [46]. This illustrates the importance of maintaining a continuous patient-GP relationship in preventing potentially inappropriate initiation of psychotropic medicines [45, 46]. The prescribing practices in our study likely reflect the considerable focus placed on limiting excessive psychotropic drugs among PwD in recent years, underscoring that the continuous deprescribing process is more than simply drug withdrawal [47].

Our data imply that the GPs conducted medication reviews based on their discretion concerning whether an evaluation would benefit the patient. Better interaction within primary care has been warranted for home-dwelling PwD, as an 18-month-long prospective study (*n*=599) showed that PwD consulted their GPs less often than other elderly persons receiving municipal health and social care services in Norway between 2009 and 2012 [48]. The national guideline for dementia strongly advises GPs to invite patients with dementia for routine checkups once or twice yearly to evaluate the need for medication reviews [6], and the GPs are reimbursed accordingly. We now demonstrate that GPs conduct medication reviews frequently (42%) and even more so when encouraged by the coordinators in LIVE@Home.Path (66%). This is in contrast to the 3.4% of consultations with patients over the age of 67 at GP level, coded as ‘medication review’ in the Norwegian Registry for Primary Health Care (NRPHC) of 2020 [49]. Of note, NRPHC does probably not catch all medication reviews in routine ambulatory GP care due to restrictions on use of reimbursement code combinations, nor contain complementary information on reasons, diagnoses, or outcomes. Additionally, the medication review reimbursement code accommodates specific formal requirements, unlike the reporting in our trial and direct comparisons can therefore not be made. Nonetheless, our findings align with a recent pragmatic prospective non-randomized intervention study confirming GPs’ preparedness to conduct medication reviews, as three peer group meetings increased the frequency of revisions and improved prescription practice, both according to the GPs themselves and the process measures in NRPHC and the Norwegian Prescription Database [50]. In our trial, the electronic medical record infrastructure was crucial to enabling collaboration and engagement between PwD and formal and informal caregivers. Our findings are uplifting in that they show that GPs now readily optimize their patients’ medications resulting in enhanced communication.

Even though we report BPSD levels close to what is reported at admission to nursing homes [12], earlier work shows that prescription rates of antidepressants, antipsychotics, anxiolytics, sedatives, and hypnotics persistently increase during the first 6 months stay [12, 46, 51]. In our study, the use of these medications was not associated with dropout due to nursing home admission, while on the contrary, impaired functioning, dependency in activities of daily living, and BPSD were associated with nursing home admission. The prospective DemVest study highlighted the pertinence of detecting and treating BPSD, as the 5-year course of these symptoms predicted functional deterioration independent of cognition in patients diagnosed with Alzheimer’s disease and Lewy body dementia [4]. Further, benzodiazepines and Z-hypnotics exacerbated functional deterioration in this cohort of 196 patients, especially when combined with antidepressants [8]. In the multicomponent cluster randomized controlled COSMOS trial (*n*=428), we documented an improvement in activities of daily living in nursing home residents after careful withdrawal of psychotropic drugs, as decided by the physician in discussion with colleagues [17, 18]. Within the intervention group of our current study, the GPs prioritized their patients for revisions according to symptoms likely to compromise safety, higher numbers of psychotropic drugs prescribed, and lower level of functioning. Our interpretation is that the GPs acknowledge the need for revisions but that a limited facility to monitor clinical change makes them more conservative when adjusting prescriptions in the home-dwelling setting compared to institutions. Another point is that inherent prescribing procedures within the multidose dispensing system, which provides machine-dispensed tablets and capsules in disposable plastic bags to patients experiencing difficulties handling and administering drugs, may increase practical challenges with changing drug regimes. Further, the fragmented organizational structure of health care services may hamper collaboration between providers, health care professionals, and PwD and their informal caregivers. As our informal caregivers to home-dwelling PwD verified the experiences from nursing homes that medication reviews improved communication between health personnel, patients, and their relatives [17], we advocate that it should be encouraged for PwD, regardless of the level of functioning and accommodation.

As this substudy concentrates on psychotropic prescribing practices, we considered medication reviews the most active ingredient within the intervention because the GPs can effectuate drug changes immediately. However, we acknowledge that it may be challenging to tease out the effects of single elements in multicomponent interventions and cannot exclude that the other components may exert more delayed effects on deprescribing [15, 16, 18, 22]. For example, increasing the dyads’ knowledge of dementia management (i.e., the Learning component) could improve symptom awareness and strategies for non-pharmacological treatment of BPSD, thereby reducing the need for psychotropic drugs over time. However, we argue that with the regular GP scheme, the dyads are at higher readiness for medication reviews than for adopting the other and less familiar components of the intervention. Effective implementation in trials and real-world settings is highly dependent on contextual factors [15]. In the above example, the intention, initial decision, and commitment to attend the learning activity represent barriers [15, 52]. To evaluate implementation in our trial, we compared the reach of medication reviews across groups. Yet, applying measures of implementation outcomes could have aided us in answering questions around fidelity and quality of implementation, mechanisms of change, and context [15, 22, 52]. Notably, due to COVID-19, the process evaluation is not completed for the trial at the time of writing [19], as the final conference where we will inquire about the coordinators and other stakeholders’ experiences is postponed. Nevertheless, a strong stakeholder incentive exists to promote the LIVE components in routine dementia care practice at present [24].

The primary strength of this study is that the participants completed assessments compiling validated, well-established and complementary instruments that were blindly and electronically administered by trained and supervised data collectors, securing data quality [19]. A considerable number of dyads were included from multiple sites and levels of health care services in Norway, thereby increasing the generalizability of our findings. The stepped-wedge design of LIVE@Home.Path was chosen in compliance with patient and public involvement, as it respects the randomization principle yet allows all participants to receive the intervention. This likely also led to low dropout rates due to withdrawal of consent.

We met COVID-19 related limitations in conducting this study. Due to the dramatically worsening care situation resulting in exacerbating BPSD and impinged trial protocol [23, 53, 54], we found it appropriate to solely analyze the prepandemic data from the first 6-month period despite compromising power and misbalancing group sizes (Fig. 1). Additionally, 19 dyads were assessed by phone rather than in person due to the outbreak, possibly lessening data quality regarding drugs and BPSD in these dyads.

This study additionally has non-COVID-19-related limitations. Firstly, our study sample was a convenience sample, and the non-random recruitment of dyads from health care services may limit the generalizability of our findings to people living with dementia somehow attended to and supported by formal and informal caregivers. Secondly, self-reports on medication may limit direct comparisons to other studies relying on data from medical records and registries. Our access was only sufficient to verify current drug consumption, and consequently, we did not inquire for prescriber details, indications, and duration of therapy. Thirdly, we did not explore the GPs’ strategies for conducting medication reviews or evaluating drug therapy, or whether they involved other health care professionals. However, the risk that the GPs to PwD allocated to the control conditions altered their behavior (i.e., increase the frequency of revisions) when being studied is minuscule as they were not yet informed on participation. Fourthly, we did not provide the GPs with formalized collegial support, or integrations for decision support other than the reports and coordinators’ involvement, and we cannot exclude the possibility that a more formalized and rigorous medication review would have yielded a greater reduction in psychotropic drug use. This pragmatic approach likely increases the variability, yet increases the external validity, of our study. Finally, the chance of false-positive findings in the subgroup analyses increases due to multiple testing.

## Conclusions

Even though psychotropic drug use and BPSD were not affected by the multicomponent intervention, our study shows that patient-GP communication improved with medication review. Implementing medication reviews in routine care could achieve long-term benefits by increasing the continuity of care for this complex patient population. We advise GPs to conduct medication reviews regularly for patients with dementia, even when prescription and follow-up are within current standards; and suggest that they, if possible, should exercise collegial support in their local networks. We recommend that future studies explore medication reviews from the GP perspective to develop integrations for decision support in dementia care.

## Supplementary Information

**Additional file 1.** The role of health care professionals involved in the conduction of the LIVE@Home.Path trial. Description: table.

**Additional file 2. **Baseline characteristics for people with dementia by medication review in the first intervention group of LIVE@Home.Path. Description: table.

**Additional file 3. **Baseline characteristics for people with dementia by attrition during the first 6-month period of LIVE@Home.Path. Description: table.

## Data Availability

The datasets used and/or analyzed during the current study are available from the corresponding author on reasonable request.

## Abbreviations

ATC: Anatomical Therapeutic Index
BPSD: Behavioral and psychological symptoms of dementia
CGIC: Clinical Global Impression of Change
COVID-19: Coronavirus SARS CoV-2 disease, 2019
CSDD: Cornell Scale for Depression in Dementia
FAST: Functional Assessment Staging
GMHR: General Medical Health Rating Scale
GP: General practitioner
IADL: Instrumental Activities of Daily Living
ICD-10: International Classification of Diseases, 10th version
LIVE: Learning; Innovation; Volunteer support; Empowerment
MMSE: Mini-Mental Status Examination
NPI-12: Neuropsychiatric Inventory, 12-domain version
NRPHC: Norwegian Registry for Primary Health Care
PSMS: Physical Self-Maintenance Scale
PwD: People with dementia

## References

[1] World Health Organization. Dementia fact sheet. World Health Organization. 2021. https://www.who.int/news-room/fact-sheets/detail/dementia. Accessed 18 Jan 2022.

[2] Vik-Mo AO, Giil LM, Borda MG, Ballard C, Aarsland D (2020). The individual course of neuropsychiatric symptoms in people with Alzheimer's and Lewy body dementia: 12-year longitudinal cohort study. Br J Psychiatry..

[3] Wergeland JN, Selbaek G, Bergh S, Soederhamn U, Kirkevold Ø (2015). Predictors for Nursing Home Admission and Death among Community-Dwelling People 70 Years and Older Who Receive Domiciliary Care. Dement Geriatr Cogn Disord..

[4] Borda MG, Aarsland D, Tovar-Rios DA, Giil LM, Ballard C, Gonzalez MC (2020). Neuropsychiatric Symptoms and Functional Decline in Alzheimerʼs Disease and Lewy Body Dementia. J Am Geriatr Soc.

[5] Bessey LJ, Walaszek A (2019). Management of Behavioral and Psychological Symptoms of Dementia. Curr Psychiatry Rep..

[6] Helsedirektoratet. Nasjonal faglig retningslinje for demens [National professional guidelines on dementia]. Helsedirektoratet. 2017; https://www.helsedirektoratet.no/retningslinjer/demens. Accessed 8 Mar 2020.

[7] Watt JA, Goodarzi Z, Veroniki AA, Nincic V, Khan PA, Ghassemi M (2020). Safety of pharmacologic interventions for neuropsychiatric symptoms in dementia: a systematic review and network meta-analysis. BMC Geriatr..

[8] Borda MG, Jaramillo-Jimenez A, Oesterhus R, Santacruz JM, Tovar-Rios DA, Soennesyn H (2021). Benzodiazepines and antidepressants: Effects on cognitive and functional decline in Alzheimer's disease and Lewy body dementia. Int J Geriatr Psychiatry..

[9] Richardson K, Loke YK, Fox C, Maidment I, Howard R, Steel N (2020). Adverse effects of Z-drugs for sleep disturbance in people living with dementia: a population-based cohort study. BMC Med..

[10] Bargagli AM, Cascini S, Agabiti N, Kirchmayer U, Marino C, Davoli M (2019). Determinants Of Antipsychotic Drugs Prescription Among Community-Living Older Adults With Dementia: A Population-Based Study Using Health Information Systems In The Lazio Region. Italy. Clin Interv Aging..

[11] Sundseth AC, Gjelstad S, Straand J, Rosvold EO (2018). General practitioners’ prescriptions of benzodiazepines, Z-hypnotics and opioid analgesics for elderly patients during direct and indirect contacts. A cross-sectional, observational study. Scand J Prim Health Care..

[12] Callegari E, Benth JŠ, Selbæk G, Lic CG, Bergh S (2021). Does Psychotropic Drug Prescription Change in Nursing Home Patients the First 6 Months After Admission?. J Am Med Dir Assoc..

[13] Strand BH, Vollrath MEMT, Skirbekk VF (2018). Dementia.

[14] Sawan MJ, Moga DC, Ma JM, Ng JC, Johnell K, Gnjidic D (2021). The value of deprescribing in older adults with dementia: a narrative review. Expert Rev Clin Pharmacol..

[15] Skivington K, Matthews L, Simpson SA, Craig P, Baird J, Blazeby JM (2021). A new framework for developing and evaluating complex interventions: an update of Medical Research Council guideance. BMJ..

[16] Ballard C, Orrell M, YongZhong S, Moniz-Cook E, Stafford J, Whittaker R (2016). Impact of antipsychotic review and nonpharmacological intervention on antipsychotic use, neuropsychiatric symptoms, and mortality in people with dementia living in nursing homes: a factorial cluster-randomized controlled trial by the well-being and health for people with dementia (WHELD) program. Am J Psychiatry..

[17] Gulla C, Flo E, Kjome RLS, Husebo BS (2019). Implementing a novel strategy for interprofessional medication review using collegial mentoring and systematic clinical evaluation in nursing homes (COSMOS). BMC Geriatr..

[18] Gedde MH, Husebo BS, Mannseth J, Kjome RLS, Naik M, Berge LI (2020). Less is more: The Impact of Deprescribing Psychotropic Drugs on Behavioral and Psychological Symptoms and Daily Functioning in Nursing Home Patients. Results from the Cluster-Randomized Controlled COSMOS Trial. Am J Geriatr Psychiatry..

[19] Husebo BS, Allore H, Achterberg W, Angeles R, Ballard BF (2020). LIVE@Home.Path—innovating the clinical pathway for home-dwelling people with dementia and their caregivers: study protocol for a mixed-method, stepped-wedge, randomized controlled trial. Trials..

[20] Wimo A, Gustavsson A, Jönsson L (2013). Application of Resource Utilization in Dementia (RUD) instrument in a global setting. Alzheimers Dement..

[21] Schulz R (2003). End-of-life care and the effects of bereavement on family caregivers of persons with dementia. N Engl J Med..

[22] Hemming K, Taljaard M, McKenzie JE, Hooper R, Copas A, Thompson JA (2018). Reporting of stepped wedge cluster randomised trials: extension of the CONSORT 2010 statement with explanation and elaboration. BMJ..

[23] Gedde MH, Husebo BS, Erdal A, Paschitz N, Vislapuu M, Angeles RC (2020). Access to and interest in assistive technology for home-dwelling people with dementia during the COVID-19 pandemic (PAN.DEM). Int Rev Psychiatry..

[24] Ministry of Health and Care Services (2015). The Dementia Plan 2020.

[25] Fæø SE, Tranvåg O, Samdal R, Husebo BS, Bruvik FK (2020). The compound role of a coordinator for home-dwelling persons with dementia and their informal caregivers: qualitative study. BMC Health Serv Res..

[26] Ulstein I, Wyller TB, Engedal K (2007). The relative stress scale, a useful instrument to identify various aspects of carer burden in dementia?. Int J Geriatr Psychiatry..

[27] Folstein MF, Folstein SE, McHugh PR (1975). Mini-Mental State - Practical Method for Grading Cognitive State of Patients for Clinician. J Psychiatr Res..

[28] Reisberg B (1988). Functional assessment staging (FAST). Psychopharmacol Bull..

[29] ATC/DDD Index. WHO collaborating Centre for Drug Statistics Methodology, Norwegian Institute of Public Health, Oslo. 2015. https://www.whocc.no/atc_ddd_index/. Accessed 01 June 2019.

[30] Cummings J (2020). The Neuropsychiatric Inventory: Development and Applications. J Geriatr Psychiatry Neurol..

[31] Aalten P, de Vugt ME, Lousberg R, Korten E, Jaspers N, Senden B (2003). Behavioral Problems in Dementia: A Factor Analysis of the Neuropsychiatric Inventory. Dement Geriatr Cogn Disord..

[32] Alexopoulos GS, Abrams RC, Young RC, Shamoian CA (1988). Cornell Scale for Depression in Dementia. Biol Psychiatry..

[33] Barca ML, Engedal K, Selbæk G (2010). A Reliability and Validity Study of the Cornell Scale among Elderly Inpatients, Using Various Clinical Criteria. Dement Geriatr Cogn Disord..

[34] Selbaek G, Kirkevold O, Sommer OH, Engedal K (2008). The reliability and validity of the Norwegian version of the neuropsychiatric inventory, nursing home version (NPI-NH). Int Psychogeriatr..

[35] Guy W. ECDEU assessment manual for psychopharmacology. Rockville: US Department of Health, Education and Welfare; 1976.

[36] Aasmul I, Husebo BS, Sampson EL, Flo E (2018). Advance Care Planning in Nursing Homes – Improving the Communication Among Patient, Family, and Staff: Results From a Cluster Randomized Controlled Trial (COSMOS). Front. Psychol..

[37] World Health Organization (1992). The ICD-10 classification of mental and behavioural disorders: clinical descriptions and diagnostic guidelines.

[38] Lawton MP, Brody EM (1969). Assessment of older people: self-maintaining and instrumental activities of daily living. Gerontologist..

[39] Lawton MP (1990). Aging and performance of home tasks. Hum Factors..

[40] Lyketsos CG, Galik E, Steele C, Steinberg M, Rosenblatt A, Warren A, et al. The General Medical Health Rating: a bedside global rating of medical comorbidity in patients with dementia. J Am Geriatr Soc 1999;47:487-491. 10.1111/j.1532-5415.1999.tb07245.x 10203127

[41] Mesquida MM, Casas MT, Sisó AF, Muñoz IG, Vian ÓH, Monserrat PT (2019). Consensus and evidence-based medication review to optimize and potentially reduce psychotropic drug prescription in institutionalized dementia patients. BMC Geriatr..

[42] Thyrian JR, Hertel J, Wucherer D, Eichler T, Michalowsky B, Dreier-Wolfgramm A (2017). Effectiveness and Safety of Dementia Care Management in Primary Care: A Randomized Clinical Trial. JAMA Psychiatry..

[43] Rikala M, Korhonen MJ, Sulkava R, Hartikainen S (2011). The effects of medication assessment on psychotropic drug use in the community-dwelling elderly. Int Psychogeriatr..

[44] Romskaug R, Skovlund E, Straand J, Molden E, Kersten H, Pitkala KH (2019). Effect of Clinical Geriatric Assessments and Collaborative Medication Reviews by Geriatrician and Family Physician for Improving Health-Related Quality of Life in Home-Dwelling Older Patients Receiving Polypharmacy: A Cluster Randomized Clinical Trial. JAMA Intern Med..

[45] Delgado J, Evans PH, Gray DP, Sidaway-Lee K, Allan L, et al. Continuity of GP care for patients with dementia: impact on prescribing and the health of patients. Br J Gen Pract. 2022. 10.3399/BJGP.2021.0413. 10.3399/BJGP.2021.0413 PMC8803082 35074796

[46] Welberry HJ, Jorm LR, Schaffer AL, Barbieri S, Hsu B, Harris MF (2021). Psychotropic medicine prescribing and polypharmacy for people with dementia entering residential aged care: the influence of changing general practitioners. Med J Aust..

[47] Reeve E, Gnjidic D, Long J, Hilmer S (2015). A systematic review of the emerging definition of ‘deprescribing’with network analysis: implications for future research and clinical practice. Br J Clin Pharmacol..

[48] Ydstebø A, Bergh S, Selbæk G, Benth JŠ, Lurås H, Vossius C (2015). The impact of dementia on the use of general practitioners among the elderly in Norway. Scand J Prim Health Care..

[49] Legemiddelgjennomgang utført av fastleger [Medication reviews conducted by general practitioners]. Helsedirektoratet, Oslo. 2021. https://www.helsedirektoratet.no/statistikk/statistikk-om-allmennlegetjenester/legemiddelgjennomgang-utfort-av-fastleger. Accessed 14 Apr 2021.

[50] Øyane NMF, Finckenhagen M, Ruths S, Thue G, Lindahl AK (2021). Improving drug prescription in general practice using a novel quality improvement model. Scand J Prim Health Care..

[51] O'Connor DW, Griffith J, McSweeney K (2010). Changes to psychotropic medications in the six months after admission to nursing homes in Melbourne Australia. Int. Psychogeriatr..

[52] Mettert K, Lewis C, Dorsey V, Halko H, Weiner B (2020). Measuring implementation outcomes: An updated systematic review of measures psychometric properties. Implementation Res. and Prac..

[53] Vislapuu M, Angeles RC, Berge LI, Kjerstad E, Gedde MH, Husebo B (2021). The consequences of COVID-19 lockdown for formal and informal resource utilization among home-dwelling people with dementia: results from the prospective PAN.DEM study. BMC Health Serv Res..

[54] Gedde MH, Husebo BS, Vahia IV, Mannseth J, Vislapuu M, Naik M (2022). The impact of COVID-19 restrictions on behavioural and psychological symptoms in home-dwelling people with dementia: a prospective cohort study (PAN.DEM). BMJ Open..

