# Supplementary material for: The impact of medication reviews by general practitioners on psychotropic drug use and behavioral and psychological symptoms in home-dwelling people with dementia: results from the multicomponent cluster randomized controlled LIVE@Home.Path trial
Source: BMC Med. 2022 May 26;20:186. doi: 10.1186/s12916-022-02382-5 (PMC9132600; doi:10.1186/s12916-022-02382-5)
Supplement: Supplementary file 1 — Additional file 1. The role of health care professionals involved in the conduction of the LIVE@Home.Path trial. Description: table. [file 12916_2022_2382_MOESM1_ESM.docx]

| **Additional file 1: The role of health care professionals involved in the conduction of the LIVE@Home.Path trial.** | |
| --- | --- |
| **Coordinators** (nurses, learning disability nurses, and occupational therapists) | Implemented the multicomponent intervention comprising Learning, Innovation, Volunteers, and Empowerment (outlined in Table 1). |
|  | Established contact with the person with dementia’s regular general practitioners to inform on trial participation and provide a report on behavioral and psychological symptoms of dementia, cognition, blood pressure, pulse, body mass index, pain, and caregiver burden (utilizing data from the data collection). |
|  | If a medication review was welcomed by the dyads, the coordinators requested it directly from the general practitioner and were encouraged to partake. |
| **The person with dementia’s general practitioner** | Conducted medication review. |
|  | Conducted advanced care planning. |
| **Researchers** | Held two-day implementation seminars qualifying the coordinators to facilitate and adapt the intervention to the dyad’s needs through lectures, role-plays, and discussions. |
|  | Provided the coordinators with guidelines, pocket manuals, checklists, developed to secure the implementation process. |
|  | Contacted the coordinators by telephone every fortnight to keep track of the implementation process. |
|  | Arranged one-day midway seminars for the coordinators to standardize and secure implementation. These midway seminars were crucial for the process evaluation. |
|  | Trained, supervised, and supported the data collectors in collecting data. |
|  | Collected data (blinded to sequence allocation). |
| **Data collectors** (nurses, learning disability nurses, occupational therapists) | Collected data (blinded to sequence allocation). |
