# Supplementary material for: The impact of medication reviews by general practitioners on psychotropic drug use and behavioral and psychological symptoms in home-dwelling people with dementia: results from the multicomponent cluster randomized controlled LIVE@Home.Path trial
Source: BMC Med. 2022 May 26;20:186. doi: 10.1186/s12916-022-02382-5 (PMC9132600; doi:10.1186/s12916-022-02382-5)
Supplement: Supplementary file 2 — Additional file 2. Baseline characteristics for people with dementia by medication review in the first intervention group of LIVE@Home.Path. Description: table. [file 12916_2022_2382_MOESM2_ESM.docx]

| **Additional file 2: Baseline characteristics for people with dementia by medication review in the intervention group of LIVE@Home.Path.** | | | | | | | |
| --- | --- | --- | --- | --- | --- | --- | --- |
|  | | | Medication review conducted  (n=44) | | No medication review conducted (n=21) | | P |
|  | | | n (%) | mean (SD)/ median [IQR] | n (%) | mean (SD)/  median [IQR] |  |
| Age | | |  | 83 (7) |  | 85 (6) | 0.206 |
| Gender, female | | | 31 (70) |  | 13 (62) |  | 0.491 |
| MMSE | | |  | 21 [18.5, 24] |  | 22 [19, 24] | 0.285 |
| FAST | | |  | 4 [4, 5] |  | 4 [4, 4] | 0.003* |
| GMHR | | |  |  |  |  | 0.653 |
|  | | Fair health | 18 (41) |  | 11 (52) |  |  |
|  | | Good health | 18 (41) |  | 8 (40) |  |  |
|  | | Excellent health | 7 (16) |  | 2 (10) |  |  |
| PSMS | | |  | 11 [8, 13] |  | 9 [7.5, 12] | 0.121 |
| IADL | | |  | 20 [16, 25] |  | 20 [15, 23] | 0.328 |
| Drugs in general | | |  |  |  |  |  |
|  | Total number | |  | 5 [3, 7] |  | 5 [4, 7] | 0.546 |
|  | Regularly | |  | 5 [2, 7] |  | 5 [3, 7] | 0.560 |
| Psychotropic drugs | | |  |  |  |  |  |
|  | Total number | |  | 1 [1, 2] |  | 1 [0, 1] | 0.046* |
|  | Regularly | |  | 1 [0, 1] |  | 1 [0, 1] | 0.180 |
|  |  | Antipsychotic drugs | 4 (9) |  | 0 (0) |  | 0.149 |
|  |  | Anxiolytic drugs | 1 (2) |  | 1 (5) |  | 0.599 |
|  |  | Hypnotic/sedative drugs | 5 (11) |  | 2 (5) |  | 0.800 |
|  |  | Antidepressant drugs | 6 (14) |  | 2 (10) |  | 0.615 |
|  |  | Anti-dementia drugs | 24 (55) |  | 8 (38) |  | 0.305 |
|  | Regularly except for anti-dementia drugs | |  | 0 [0, 1] |  | 0 [0, 0] | 0.507 |
|  | On-demand | |  | 0 [0, 0] |  | 0 [0, 0] | 0.457 |
|  |  | Anxiolytic drugs | 1 (2) |  | 1 (5) |  | 0.599 |
|  |  | Hypnotic/sedative drugs | 6 (14) |  | 0 (0) |  | 0.072 |
| NPI-12 total score | | |  | 18 [6, 29] |  | 14 [3, 20] | 0.081 |
| NPI-12 subsyndromes | | |  |  |  |  |  |
|  | | Psychosis |  | 0 [0, 2] |  | 0 [0, 2] | 0.151 |
|  | | Hyperactive behavior |  | 2 [0, 8] |  | 2 [0, 5] | 0.845 |
|  | | Mood |  | 7 [1, 14] |  | 4.5 [0, 11] | 0.414 |
| NPI-12 domain scores | | |  |  |  |  |  |
|  | | Delusions |  | 0 [0, 2] |  | 0 [0, 1] | 0.298 |
|  | | Hallucinations |  | 0 [0, 2] |  | 0 [0, 0] | 0.040* |
|  | | Agitation |  | 0 [0, 1] |  | 0 [0, 0] | 0.004* |
|  | | Depression |  | 1 [0, 6] |  | 0 [0, 3] | 0.229 |
|  | | Anxiety |  | 0 [0, 4] |  | 0 [0, 0] | 0.125 |
|  | | Euphoria |  | 0 [0, 0] |  | 0 [0, 0] | 0.469 |
|  | | Apathy |  | 1 [0, 8] |  | 0 [0, 3] | 0.058 |
|  | | Disinhibitions |  | 0 [0, 1] |  | 0 [0, 0] | 0.434 |
|  | | Irritability |  | 0 [0, 4] |  | 0 [0, 3] | 0.600 |
|  | | Aberrant motor behavior |  | 0 [0, 3] |  | 0 [0, 0] | 0.798 |
|  | | Sleep disturbances |  | 0 [0, 3] |  | 0 [0, 0] | 0.451 |
|  | | Appetite changes |  | 0 [0, 4] |  | 0 [0, 6] | 0.798 |
| NPI-12 domains of clinical relevance | | |  | 2 [1, 4] |  | 2 [0, 2] | 0.135 |
| ≥ 1 NPI-12 domain of clinical relevance | | | 36 (82) |  | 14 (32) |  | 0.175 |
| CSDD total score | | |  | 7 [2, 9] |  | 4 [1, 7] | 0.197 |
| CSDD total score of clinical relevance | | | 17 (39) |  | 5 (24) |  | 0.169 |
| Table legends: Unknown whether medication review was conducted during the first 6-month intervention period in two participants (n) of the 67 completers at 6 months. SD: Standard deviation. P: Two-tailed P-value, generated by Pearson’s chi-square, unequal variances t-test, or Wilcoxon-Mann-Whitney test, regarded significant if <0.05 and marked *. MMSE: Mini-Mental Status Examination, range 0-30, a lower score indicates greater impairment. FAST: Functional Assessment Staging, range 1-7, a higher score indicates lesser functioning. GMHR: General Medical Health Rating Scale, a one-item, four-point scale evaluating medical comorbidity. PSMS: Physical Self-Maintenance Scale, range 6-30, a higher score indicates higher dependency. IADL: Instrumental Activities of Daily Living Scale, range 8-31, higher score indicates higher dependency. Drugs were classified by the Anatomical Therapeutic Chemical Index; psychotropic drugs included antipsychotics, anxiolytics, hypnotics/sedatives, antidepressants, and anti-dementia drugs. NPI-12: Neuropsychiatric Inventory, total score ranges 0-144, psychosis subsyndrome (delusions and hallucinations) ranges 0-24, hyperactive behavior (agitation, euphoria, irritation, disinhibition, aberrant motor behavior) ranges 0-60, mood (depression, apathy, sleep disturbances, and appetite changes) ranges 0-48, each domain ranges 0-12 with domain scores ≥4 indicating symptoms of clinical relevance. CSDD: Cornell Scale for Depression in Dementia, total score ranges 0-38 and ≥8 indicate depressive symptoms of clinical relevance. | | | | | | | |
