# Supplementary material for: The impact of medication reviews by general practitioners on psychotropic drug use and behavioral and psychological symptoms in home-dwelling people with dementia: results from the multicomponent cluster randomized controlled LIVE@Home.Path trial
Source: BMC Med. 2022 May 26;20:186. doi: 10.1186/s12916-022-02382-5 (PMC9132600; doi:10.1186/s12916-022-02382-5)
Supplement: Supplementary file 3 — Additional file 3. Baseline characteristics for people with dementia by attrition during the first 6-month period of LIVE@Home.Path. Description: table. [file 12916_2022_2382_MOESM3_ESM.docx]

| **Additional file 3: Baseline characteristics for people with dementia by attrition during the first 6-month period of LIVE@Home.Path.** | | | | | | | | | | | | | | | |
| --- | --- | --- | --- | --- | --- | --- | --- | --- | --- | --- | --- | --- | --- | --- | --- |
|  | | | | Included  (n= 280) | | | Completers  (n= 237) | | | Non-completers  (n= 43) | | | P | | |
|  | | | | n (%) | mean (SD)/ median [IQR] | | | n (%) | mean (SD)/ median [IQR] | | n (%) | mean (SD)/ median [IQR] | |  |  |
| Age | | | |  | | 82 (7) |  | | 82 (7) |  | | 85 (7) | 0.018* | | |
| Gender, female | | | | 174 (62) | |  | 149 (63) | |  | 25 (58) | |  | 0.665 | | |
| MMSE | | | |  | | 21 [18, 23] |  | | 21 [18, 23] |  | | 20 [18, 23] | 0.558 | | |
| FAST | | | |  | | 4 [4, 4] |  | | 4 [4, 4] |  | | 4 [4, 6] | 0.005* | | |
| GMHR | | | |  | |  |  | |  |  | |  | 0.633 | | |
|  | | Poor health | |  | | 7 (3) |  | | 5 (2) |  | | 2 (5) |  | | |
|  | | Fair health | |  | | 90 (32) |  | | 74 (31) |  | | 16 (37) |  | | |
|  | | Good health | |  | | 127 (45) |  | | 110 (46) |  | | 17 (40) |  | | |
|  | | Excellent health | |  | | 47 (17) |  | | 40 (17) |  | | 7 (16) |  | | |
| PSMS | | | |  | | 10 [8, 12] |  | | 10 [8, 12] |  | | 12 [9, 15] | 0.011* | | |
| IADL | | | |  | | 21 [15, 25] |  | | 20 [15, 25] |  | | 24 [16, 28] | 0.006* | | |
| Drugs in general | | | |  | |  |  | |  |  | |  |  | | |
|  | Total number | | |  | | 5 [3, 7] |  | | 5 [3, 7] |  | | 5 [3, 8] | 0.928 | | |
|  | Regularly | | |  | | 5 [3, 7] |  | | 5 [3, 7] |  | | 4 [3, 6.5] | 0.739 | | |
| Psychotropic drugs | | | |  | |  |  | |  |  | |  |  | | |
|  | Total number | | |  | | 1 [0, 1] |  | | 1 [0, 1] |  | | 1 [0, 1] | 0.084 | | |
|  | Regularly | | |  | | 1 [0, 1] |  | | 1 [0, 1] |  | | 0.5 [0, 1] | 0.067 | | |
|  |  | | Antipsychotic drugs | 14 (5) | |  | 11 (5) | |  | 3 (7) | |  | 0.483 | | |
|  |  | | Anxiolytic drugs | 5 (2) | |  | 5 (2) | |  | 0 (0) | |  | 0.344 | | |
|  |  | | Hypnotic/sedative drugs | 36 (13) | |  | 31 (13) | |  | 5 (12) | |  | 0.851 | | |
|  |  | | Antidepressant drugs | 38 (14) | |  | 31 (13) | |  | 7 (16) | |  | 0.523 | | |
|  |  | | Anti-dementia drugs | 122 (44) | |  | 112 (47) | |  | 10 (23) | |  | 0.017* | | |
|  | Regularly except for anti-dementia drugs | | |  | | 0 [0, 1] |  | | 0 [0, 1] |  | | 0 [0, 1] | 0.799 | | |
|  | On-demand | | |  | | 0 [0, 0] |  | | 0 [0, 0] |  | | 0 [0, 0] | 0.965 | | |
|  |  | | Antipsychotic drugs | 0 (0) | |  | 1 (0) | |  | 0 (0) | |  | 0.675 | | |
|  |  | | Anxiolytic drugs | 10 (4) | |  | 8 (3) | |  | 2 (5) | |  | 0.646 | | |
|  |  | | Hypnotic/sedative drugs | 11 (4) | |  | 9 (4) | |  | 2 (5) | |  | 0.757 | | |
| NPI-12 total score | | | |  | | 13 [5, 25] |  | | 12 [4, 24] |  | | 16 [8, 29] | 0.054 | | |
| NPI-12 domains of clinical relevance | | | |  | | 1 [0, 3] |  | | 1 [0, 3] |  | | 2 [1, 4] | 0.080 | | |
| CSDD total score | | | |  | | 5 [2, 9] |  | | 5 [1, 9] |  | | 6 [2, 9] | 0.470 | | |
| CSDD total score of clinical relevance | | | | 85 (30) | |  | 73 (30) | |  | 12 (28) | |  | 0.809 | | |
| Table legends: n: number of participants. SD: standard deviation. IQR: interquartile range. P: Two-tailed P-value, generated by Pearson’s chi-square, unequal variances t-test, or Wilcoxon-Mann-Whitney test, regarded significant if <0.05 and marked *. MMSE: Mini-Mental Status Examination, range 0-30, a lower score indicates greater impairment. FAST: Functional Assessment Staging, range 1-7, a higher score indicates lesser functioning. GMHR: General Medical Health Rating Scale, a one-item, four-point scale evaluating medical comorbidity. PSMS: Physical Self-Maintenance Scale, range 6-30, a higher score indicates higher dependency. IADL: Instrumental Activities of Daily Living Scale, range 8-31, higher score indicates higher dependency. Drugs were classified by the Anatomical Therapeutic Chemical Index; psychotropic drugs included antipsychotics, anxiolytics, hypnotics/sedatives, antidepressants, and anti-dementia drugs. NPI-12: Neuropsychiatric Inventory, total score ranges 0-144, each of the 12 domains ranges 0-12 with domain scores ≥4 indicating symptoms of clinical relevance. CSDD: Cornell Scale for Depression in Dementia, total score ranges 0-38 and ≥8 indicate depressive symptoms of clinical relevance. | | | | | | | | | | | | | | | |
